# The evolving HIV epidemic in Ontario, Canada: A retrospective analysis of new HIV diagnoses to identify subpopulations with persistent risk of HIV transmission

**DOI:** 10.17269/s41997-025-00997-8

**Published:** 2025-03-18

**Authors:** Abigail Kroch, David Gogolishvili, Kristen O’Brien, Lauren Orser, Kevin Woodward, Wangari Tharao, Daniel Lazzam, Katherine Burress, Majorie Kabahenda, Mona Loutfy, Patrick O’Byrne

**Affiliations:** 1 Ontario HIV Treatment Network, Toronto, ON Canada; 2 Dalla Lana School of Public Health, University of Toronto, Toronto, ON Canada; 3 Public Health Ontario, Toronto, ON Canada; 4 University of Ottawa School of Nursing, Ottawa, ON Canada; 5 Ottawa Public Health Sexual Health Clinic, Ottawa, ON Canada; 6 Division of Infectious Diseases at McMaster University, Hamilton, ON Canada; 7 Women’s Health in Women’s Hands, Toronto, ON Canada; 8 Department of Medicine, McMaster University, Hamilton, ON Canada; 9 Maple Leaf Medical Clinic, Toronto, ON Canada; 10 Women’s College Hospital, Toronto, ON Canada

**Keywords:** HIV, Surveillance, Gay men’s health, Immigration health, HIV epidemiology, VIH, Surveillance, Santé des hommes gais, Santé des immigrants, Épidémiologie du VIH

## Abstract

**Objective:**

The objective of this study was to characterize new HIV diagnoses in key Ontario cities, in order to understand current drivers of continued HIV transmission to inform HIV testing and prevention efforts.

**Methods:**

Chart reviews were carried out at four clinical sites in Ontario, Canada. The study population included individuals who were diagnosed with HIV in Ottawa, Hamilton, and Toronto between January 1, 2018, and December 31, 2020, and had no previous evidence of HIV documented.

**Results:**

The total number of persons in this analysis was 359, from Toronto (*n* = 201), Ottawa (*n* = 88), and Hamilton (*n* = 70). More than half of the diagnoses were among those who immigrated to Canada, and many were diagnosed (11%) during the year they arrived. Many participants experienced a late diagnosis (43.2%), and while 116 (32.3%) had HIV testing history in Ontario, 155 did not (43.2%). Many participants were men who have sex with men (MSM) and had a previously recorded diagnosis of gonorrhea or chlamydia (*n* = 27; 7.5%) or syphilis (*n* = 39; 10.9%). Among women and heterosexual men, a diagnosis of hepatitis C (*n* = 18; 5.0%) appeared to signal a risk of HIV diagnosis.

**Conclusion:**

These data show that HIV testing and prevention strategies should be targeted to (1) MSM with a history of syphilis, gonorrhea, or chlamydia; (2) heterosexual men and women with a history of hepatitis C; and (3) immigrants within the first 5 years of migration. To address the evolving epidemic, it will be necessary to employ targeted HIV screening and prevention measures.

## Résumé

**Objectif:**

L’objectif de cette étude était de caractériser les nouveaux diagnostics de VIH dans les principales villes de l’Ontario, afin de comprendre les facteurs liés à la transmission continue du VIH et d’informer les efforts de dépistage et de prévention du VIH.

**Méthodes:**

Des examens de dossiers ont été effectués dans quatre sites cliniques de l’Ontario, au Canada. La population étudiée comprenait les personnes qui ont été diagnostiquées avec le VIH à Ottawa, Hamilton et Toronto entre le 1er janvier 2018 et le 31 décembre 2020 et qui n’avaient aucune preuve antérieure de VIH documentée.

**Résultats:**

Le nombre total de personnes dans cette analyse était de 359, provenant de Toronto (*n* = 201), Ottawa (*n* = 88) et Hamilton (*n* = 70). Plus de la moitié des diagnostics ont été posés par des personnes ayant immigré au Canada, et un grand nombre d’entre elles ont été diagnostiquées (11%) au cours de l’année de leur arrivée. Un nombre non négligeable de participants ont été diagnostiqués tardivement (43,2%), et si 116 (32,3%) avaient des antécédents de dépistage du VIH en Ontario, 155 n’en avaient pas (43,2%). Un grand nombre de participants étaient des hommes ayant des relations sexuelles avec d’autres hommes (HSH) et avaient déjà reçu un diagnostic de gonorrhée ou de chlamydia (*n* = 27; 7,5%) ou de syphilis (*n* = 39; 10,9%). Parmi les femmes et les hommes hétérosexuels, un diagnostic d’hépatite C (*n* = 18; 5,0%) semblait signaler un risque de diagnostic du VIH.

**Conclusion:**

Ces données montrent que les stratégies de dépistage et de prévention du VIH devraient être ciblées sur: 1) les HSH ayant des antécédents de syphilis, de gonorrhée ou de chlamydia, 2) les femmes et les hommes hétérosexuels ayant des antécédents d’hépatite C et 3) les immigrants au cours des cinq premières années de leur migration. Pour faire face à l’évolution de l’épidémie, il sera nécessaire de recourir à des mesures ciblées de dépistage et de prévention du VIH.

## Introduction

In 2020, the UNAIDS updated its global targets to 95-95-95, signalling that 95% of persons living with HIV are diagnosed, that 95% of those who are diagnosed are engaged in care, and that 95% of those in care achieve a suppressed viral load (and thus cannot transmit the virus to sexual partners). A key item within these 95-95-95 targets is that they apply to all sub-populations of persons affected by HIV. This highlights a strong need to better understand HIV transmission and diagnosis, impacts of prevention strategies, and linkage to care within these sub-groups.

In Ontario, from 2018 to 2020, 55% of new HIV diagnoses were among men who have sex with men (MSM), 12% were among people who use injection drugs, 27% were among African, Caribbean, and Black people, and 22% were among women (OHESI, 2022a). In each population, differing access to HIV testing and prevention services, plus varying risk factors, yields a complex situation consisting of multiple simultaneous epidemics. This provincial context is further complicated, as it is in many high-income countries, by high immigration rates from countries with an elevated prevalence of HIV and asylum seekers from 2SLGBTQ + communities. Therefore, to assess drivers of diagnosis and ongoing transmission, we must expand beyond typical surveillance metrics to determine how to intervene in populations that do not regularly present for HIV testing.

To generate some nascent data on this topic, we set out to better characterize new diagnoses in key Ontario cities, in order to understand predictors of HIV transmission and further identify key populations for interventions for HIV testing and prevention efforts. In particular, we examined three key pieces of information: (1) immigration history, (2) history of sexually transmitted and bloodborne infections (STBBIs), and (3) access to HIV testing through Ontario test history and late diagnosis metrics. Our rationale for focusing on these data was multipart.

First, we focused our analysis on immigration because it contributes multiple complex factors to understanding HIV transmission in Ontario. For one, immigration leads to a shifting population with growth in the demographics of people at risk for HIV. As well, because public health surveillance data are often incomplete, there are many diagnoses for those who already know their status and who are entering HIV care post-migration. Next, exposure to HIV may occur prior to migration and, though people learn their HIV-positive status in Ontario, acquisition of infection may have occurred elsewhere. Within Canada, surveillance reporting varies with respect to the determination of diagnoses among those who had previously received an HIV diagnosis from another province, territory, or country, relative to those who had received a new HIV diagnosis within the province and are learning their status for the first time (Popovic et al., 2019). The Public Health Agency of Canada (PHAC) recently assessed this potential double reporting of new HIV cases after noting a rise in new HIV cases in Canada between 2014 and 2017, and found that five provinces and territories included cases previously diagnosed in other Canadian provinces or territories within the HIV surveillance data reported to PHAC; additionally, nine provinces and territories included people who were diagnosed with HIV outside of Canada (Popovic et al., 2019). The Ontario HIV Epidemiological Surveillance Initiative (OHESI) typically reports on previous evidence of HIV, with 22% of diagnoses from 2018 to 2020 among those who knew their status at the time of testing (OHESI, 2022a).

Second, our rationale for examining STBBI diagnoses before an HIV diagnosis was that, internationally, there is clear evidence that syphilis is associated with an increased risk of sexual acquisition and transmission of HIV. For example, the 2014 Preexposure Prophylaxis (PrEP) Initiative (iPrEx) study of 2499 HIV-seronegative men and transgender women who have sex with men found that HIV incidence varied by incident syphilis (2.8 cases per 100 person-years for no syphilis vs 8.0 cases per 100 person-years for incident syphilis), reflecting a hazard ratio of 2.6 (95%CI 1.6–4.4; *p* < 0.001) for those with syphilis (Solomon et al., 2014). A 2021 systematic review and meta-analysis of 22 studies involving 65,232 participants from across the world found that syphilis infection almost tripled the risk of HIV acquisition (Wu et al., 2021). The results and the magnitude of the associations for other high-risk populations (female sex workers, serodiscordant couples, people who inject drugs, and attendees of STI clinics) were similar to those for MSM (RR 2.98, 95%CI 2.15–4.14; vs RR 2.60, 95%CI 1.78–3.80, respectively), and there was no evidence to suggest MSM were at greater risk than other high-risk populations (Wu et al., 2021). There is emerging evidence that gonorrhea incidence is also associated with HIV among MSM. A review of eight studies found a direct strong correlation (Pearson correlation coefficient 0.94 [95%CI, 0.68–0.99]) between the incidence of rectal gonorrhea and HIV infection in MSM who were not using PrEP (Mullick & Murray, 2020). Moreover, in the United States, about 25% of people living with HIV are coinfected with hepatitis C virus (HCV), and nearly 75% of people with HIV who inject drugs are also infected with HCV (CDC, 2017). Similarly, it is estimated in Canada that about 20% of the HIV-positive population are coinfected with HCV (Hull et al., 2016). The highest rates of coinfection in Canada are found among people who inject drugs; however, increasing outbreaks of sexually transmitted HCV, usually in the context of drug use, occur among MSM, especially those who are living with HIV (CANHepC, 2019). According to a 2016 modelling study, HCV prevalence can be used as a proxy biomarker of HIV epidemic potential among people who inject drugs, where the scale and evolution of HIV epidemic expansion can be predicted based on HCV diagnoses (Akbarzadeh et al., 2016).

Finally, while the use of PrEP has led to decreases in HIV diagnoses among MSM in many jurisdictions, other sub-populations have not experienced the same decrease (Grulich & Bavinton, 2022; Kroch et al., 2023). Examination of HIV testing history and the stage of HIV infection at the time of diagnosis can help us better understand how to intervene with people from other HIV sub-populations who may have missed opportunities for PrEP and testing. Having a low CD4 cell count, often defined as < 350 cells/mm^3^, at the time of HIV diagnosis is commonly referred to as late diagnosis in high-income countries (van Opstal et al., 2018). A 2014 systematic review reported that across 33 high-income countries with data between 2006 and 2011, a median of 29% (range 17–50%) of HIV diagnoses were considered late based on locally defined parameters (Sullivan et al., 2014). A 2010 study by Althoff et al. (2010) assessed the immune status of 44,491 patients at initial presentation for HIV care from 1997 to 2007 in 13 US and Canadian clinical cohorts. These authors found that CD4 count at first presentation for HIV care has increased annually over this time period but has remained < 350 cells/mm^3^, suggesting the urgent need for earlier HIV diagnosis and treatment (Althoff et al., 2010). Similarly, in a group of 1819 study participants who were newly diagnosed in Ontario from 1999 to 2013, 53% had a late diagnosis and 54% were late presenters (Wilton et al., 2019); this was similarly identified in Ottawa, Ontario, between 2015 and 2021 (Orser et al., 2022). In another study among all newly diagnosed patients between 1995 and 2010 in Southern Alberta, 59% were late presenters (Krentz & Gill, 2012). Ontario has experienced a 53% increase in testing from 2010 to 2018, with 418,200 tests in 2010 and 637,788 tests in 2018 (OHESI, 2022b). Late diagnoses in persons diagnosed with HIV have not been examined in recent years and are not regularly reported in HIV surveillance.

Taken as a whole, immigration, previous diagnoses of STBBIs, and timing of diagnosis are key factors in understanding the drivers of an evolving HIV epidemic and they can be used to inform HIV screening and prevention strategies. Therefore, the objective of this study was to characterize new HIV diagnoses in key Ontario cities, in order (1) to understand current drivers of continued HIV transmission, and (2) to inform HIV testing and prevention efforts.

## Methods

To better understand the correlates and possible drivers of HIV transmission in Ontario, Canada, chart reviews were carried out at four sites; three were clinical sites (two primary care clinics in Toronto and a sexual health clinic in Hamilton) and one was a sexual health clinic at a public health unit (Ottawa). These clinics serve populations of people at risk for HIV and sexually transmitted infections, and the patients included in this study attended the clinic for HIV testing or were referred to the clinic due to their HIV status. These sites represent a diversity of geography and patient characteristics. Trained data collectors conducted chart reviews with respect to standardized indicators. Inclusion criteria included individuals residing in Ottawa, Hamilton, and Toronto who were diagnosed with HIV and previously unaware of their diagnosis (i.e., first-time diagnosis) between January 1, 2018, and December 31, 2020. Individuals with a positive HIV test who reported or were found to have had a prior HIV diagnosis in another province, territory, or country were excluded from this analysis.

Manual review of charts was required, even when the data were stored in an electronic medical record. Much of this information was stored in notes and not structured fields; therefore, manual abstraction ensured greater accuracy and completion of the data. The following variables were gathered from the charts: date of HIV diagnosis, sex at birth, race/ethnicity, country of birth, year of immigration to Canada, history of HIV testing, lifetime history of STBBI diagnoses (hepatitis C (active or resolved), gonorrhea, chlamydia, syphilis) recorded in the patient chart (either through laboratory testing or patient reported in notes), HIV risk factors (MSM, injection drug use, heterosexual sex), late HIV diagnosis (defined by a CD4 count ≤ 350 cells/mm^3^), concurrent AIDS diagnosis (defined by CD4 count ≤ 200 cells/mm^3^ and/or presence of AIDS defining illness), and reason for HIV testing. Multiple HIV risk factors could be reported per participant, and no hierarchy was assigned to rank risk factors; all responses are reported. Those with AIDS diagnosis defined by their CD4 count are also included as late diagnoses. These data were extracted from lab reports and notes that were written at the person’s time of diagnosis.

Study data were collected and managed using REDCap electronic data capture tools hosted at the Ontario HIV Treatment Network (Harris et al., 2009, 2019). REDCap (Research Electronic Data Capture) is a secure, web-based software platform designed to support data capture for research studies, providing (1) an intuitive interface for validated data capture; (2) audit trails for tracking data manipulation and export procedures; (3) automated export procedures for seamless data downloads to common statistical packages; and (4) procedures for data integration and interoperability with external sources. One site provided an extract of the electronic medical record and an enhanced review of charts using Microsoft Excel. All data were aggregated and cleaned using SAS v.9.5 and all analyses were carried out in SAS. Univariate and bivariate tables are provided, with chi-square analyses where appropriate. A *p*-value of 0.05 was selected a priori to determine statistical significance.

This study was covered by research ethics at University of Toronto for the multi-site study (PI Kroch, REB 00041229), as well as site-specific ethics applications at Hamilton Integrated Research Ethics Board (PI Woodward, 13784-C), University of Toronto (PI Loutfy, 00041367), and University of Ottawa (PI O’Byrne H-03–21–6786).

## Results

The total number of persons in this analysis was 359, with the majority coming from sites in Toronto (*n* = 201), followed by Ottawa (*n* = 88) and Hamilton (*n* = 70). For reference, this represents 19% of diagnoses captured in Ontario’s provincial surveillance data over this time period (OHESI, 2022a). Demographic and risk factor information are presented in Table 1. Sex at birth, race/ethnicity, and risk factors of participants were similar to those in surveillance reporting. Information not captured in typical surveillance reporting included immigration year and country of origin, with 46.8% of participants being foreign-born (born outside of Canada), with a median time of 3 years (interquartile range (IQR) arrival year, 9 years) in Canada prior to HIV diagnosis. Of those who were foreign-born, only 72 people had a known country of origin. A total of 82% of participants with a known country of origin were born in a country with a generalized HIV epidemic. The median age was 36 years (IQR 30, 47).

**Table 1:** Demographics and risk factors of study participants

	*N*	Percent
Total participants	359	
Year of diagnosis		
2018	127	35.38
2019	114	31.75
2020	118	32.87
Sex at birth		
Male	281	78.3
Female	78	21.7
Race/ethnicity		
Black	86	24
White	146	40.7
Asian	9	2.5
Latino	27	7.5
Other (Indigenous, Middle Eastern, Mixed)	7	1.9
Missing	84	23.4
Age categories		
< 25	35	9.8
25–34	119	33.2
35–44	100	27.9
45–54	55	15.3
≥ 55	50	13.9
Country of origin		
Canadian-born	136	37.9
Missing origin	80	22.3
Foreign-born	143	39.8
Arrived within 1 year	39	27.3
1 – < 5 years	61	42.7
5 – < 10 years	19	13.3
≥ 10 years	24	16.8
HIV-related risk factors		
History of injection drug use	43	12
Men who have sex with men (MSM)	202	56.2
Heterosexual sex	92	25.6

To determine whether there were missed opportunities for prevention activities, we examined testing behaviours, history of previously diagnosed STBBIs, late HIV diagnoses, and AIDS diagnoses (Table 2). Many participants experienced a late diagnosis (43.2%), although CD4 was missing for 88 participants (24.5%). While 116 (32.3%) had HIV testing history in Ontario, 155 did not (43.2%) and 88 had unknown test history (24.5%). Many participants had a previous recorded diagnosis of gonorrhea or chlamydia (*n* = 27; 7.5%), syphilis (*n* = 39; 10.9%), or hepatitis C (*n* = 18; 5.0%). The full sample was included in the denominator and is indicative of prior testing in Ontario.

**Table 2:** Lifetime HIV testing, diagnosis timing and STBBI diagnoses

	*N*	Percent
Total participants	359	
Gonorrhea/chlamydia	27	7.5
Syphilis	39	10.9
Hepatitis C	18	5.0
History of HIV testing in Ontario	116	32.3
AIDS diagnosis	83	23.1
Late HIV diagnosis	155	43.2
Missing test history/CD4 data	88	24.5

In total, 74 participants (20.6%) had at least one previous STBBI diagnosis and therefore may have missed an opportunity for HIV testing or PrEP referral. We further conducted bivariate analysis to determine these risk factors by subpopulation and the relationship between immigration status and HIV-related risk factors. In Table 3, we examine the relationship of HIV testing and STBBI diagnosis to sex at birth and MSM risk factors. Females who were diagnosed with HIV were more likely to be Black and to be diagnosed during the arrival year of immigration. MSM had higher rates of STI diagnoses (i.e., gonorrhea, chlamydia, and/or syphilis) prior to and concurrent with HIV diagnosis, were more likely to have previously had an HIV test in Ontario, and were less likely to have an AIDS diagnosis concurrent with their HIV diagnosis, whereas females and heterosexual males showed higher rates of previous diagnoses of hepatitis C.

**Table 3:** Sex and orientation by demographics, HIV testing and lifetime STBBI diagnoses

		Men who have sex with men		Heterosexual males		Females		*p* value
		*N*	%	*N*	%	*N*	%	
Race/ethnicity								< 0.0001
Black		23	11.4	18	22.8	45	57.7	
White		84	41.6	40	50.6	22	28.2	
Other (Asian, Latino, Indigenous, Middle Eastern, Mixed)		31	15.3	6	7.6	6	7.7	
Missing		64	31.7	15	19	5	6.4	
Age at diagnosis								0.0005
Median (yrs)		34		44		37		
Country of origin								< 0.0001
Canadian-born		77	38.12	34	43	25	32.1	
Missing		53	26.24	15	19	12	15.4	
Foreign-born		72	35.6	30	38	41	52.6	
Arrived within 1 year		7	9.7	11	36.7	21	51.2	
1 – < 5 years		37	51.4	8	26.7	16	39	
5 – < 10 years		14	19.4	3	10	2	4.9	
≥ 10 years		14	19.4	8	26.7	2	4.9	
Ontario HIV test history								< 0.01
No		81	40.1	41	51.9	33	42.3	
Yes		83	41.1	20	25.3	13	16.7	
Unknown		38	18.8	18	22.8	32	41	
AIDS diagnosis		202		79		78		0.0211
No		117	57.9	36	45.6	35	44.9	
Yes		39	19.3	27	34.2	17	21.8	
Unknown		46	22.8	16	20.3	26	33.3	
History of hepatitis C								< 0.0001
No		202	100	69	87.3	70	89.7	
Yes		0	0	10	12.7	8	10.3	
History of gonorrhea/chlamydia								0.0017
No		178	88.1	77	97.5	77	98.7	
Yes		24	11.9	2	2.5	1	1.3	
History of syphilis								0.0005
No		169	83.7	74	93.7	77	98.7	
Yes		33	16.3	5	6.3	1	1.3	

Understanding local HIV transmission is complicated by high immigration rates. There can be both importation of cases with known HIV-positive status, diagnosis of HIV for the first time in Ontario while transmission occurred elsewhere, and increased risk factors post-migration leading to new HIV transmissions. For this reason, we examined time in Canada post-immigration relative to missed opportunities for HIV testing or prevention (Table 4). People diagnosed with HIV post-migration were more likely to be Black or of other race/ethnicities, compared to people who were Canadian-born. More than half of diagnoses were among those who immigrated to Canada, and among them, many were diagnosed during the year they arrived (27.3%), with 84.6% of those diagnosed during their arrival year identifying as Black. Among diagnoses in Black females, 54% occurred among immigrants during their arrival year and 89% within 5 years after arrival; whereas for Black males, 38.2% of diagnoses occurred among immigrants during their arrival year, 68% within 5 years after arrival, and 15% among Canadian-born Black males.

**Table 4:** HIV testing and lifetime STBBI diagnoses by country of origin

	Canadian-born		Foreign-born								*p* value
			Arrived within 1 year		1 – < 5 years		5 – < 10 years		≥ 10 years		
	*N*	%	*N*	%	*N*	%	*N*	%	*N*	%	
Race/ethnicity											< 0.0001
Black	6	4.4	33	84.6	23	37.7	5	26.3	4	16.7	
White	113	83.1	0	0	5	8.2	3	15.8	5	20.8	
Other (Asian, Latino, Indigenous, Middle Eastern, Mixed)	3	2.2	4	10.3	19	31.1	7	36.8	4	16.7	
Missing	14	10.3	2	5.13	14	23	4	21.1	11	45.8	
Age at diagnosis (years)											
Median (IQR)	37	(30,50)	40	(33,49)	34	(29,37)	33	(29,36)	45	(37,62)	
Ontario HIV test history											< 0.0001
No	39	28.7	32	82.1	41	67.2	4	21.1	14	58.3	
Yes	64	47.1	1	2.6	7	11.5	12	63.2	4	16.7	
Unknown	33	24.3	6	15.4	13	21.3	3	15.8	6	25	
AIDS diagnosis											< 0.0001
No	106	77.9	22	56.4	30	49.2	17	89.5	15	62.5	
Yes	27	19.9	13	33.3	14	23	2	10.5	9	37.5	
Unknown	3	2.2	4	10.3	17	27.9	0	0	0	0	
History of hepatitis C											0.0008
No	119	87.5	39	100	61	100	19	100	24	100	
Yes	17	12.5	0	0	0	0	0	0	0	0	
History of gonorrhea/chlamydia											0.0053
No	119	87.5	38	97.4	60	98.4	15	78.9	24	100	
Yes	17	12.5	1	2.6	1	1.6	4	21.1	0	0	
History of syphilis											0.3084
No	116	85.3	36	92.3	57	93.4	17	89.5	23	95.8	
Yes	20	14.7	3	7.7	4	6.6	2	10.5	1	4.2	

Of course, history of HIV testing and STBBI diagnoses would have limited availability for recent immigrants. However, we do find that for both hepatitis C and gonorrhea/chlamydia, all to almost all diagnoses occurred in those who were Canadian-born. While syphilis showed a similar pattern, with the highest rate among those who were Canadian-born, a greater occurrence of syphilis diagnoses did occur in immigrants, but these differences were not statistically significant. Those diagnosed during their arrival year did have a greater percentage of AIDS diagnoses (33.3%), and interestingly those living in Canada for more than 10 years also had a higher rate of AIDS diagnoses (37.5%). Further, those living in Canada for more than 10 years also had lower rates of HIV testing in Ontario (58.3% with no Ontario test history).

## Discussion

In this retrospective study, we reviewed new HIV diagnoses at four sites in Ottawa, Hamilton, and Toronto, Ontario between January 1, 2018, and December 31, 2020. Our analysis included 359 persons (*n* = 201 from Toronto, *n* = 88 from Ottawa, and *n* = 70 from Hamilton), which represented 19% of new HIV diagnoses in the province during that time. Notably, we found that MSM were more likely to have prior STI diagnoses, that heterosexual men and women were more likely to have had prior hepatitis C diagnoses, and that most new diagnoses among Black women occurred within their first 5 years post-migration to Ontario. These findings raise a few interesting points for discussion.

First, while immigration patterns complicate the understanding of local transmission, these data elucidate that timely engagement in HIV testing is necessary for migrants, especially for MSM and those from countries with a high prevalence of HIV. Early testing will not only help identify undiagnosed infections, but also support an early introduction to prevention strategies, access to treatment, and ultimately U = U (i.e., undetectable equals untransmittable) (The Lancet HIV, 2017).

Ongoing linkage to testing and prevention services after arrival, however, is also likely needed. HIV risks may change post-migration due to changes in risk behaviours or a lack of understanding of local HIV risks. A 2018 review article notes that a high proportion of migrants acquire HIV after migration, and this group frequently presents to care late (Ross et al., 2018). Migrants living in high-income countries are disproportionately affected by HIV infection. The proportion of new HIV diagnoses among migrants exceeds the percentage of foreign-born persons in the general population in nearly all high-income countries, and is as high as 70% in some European countries (Ross et al., 2018). Prior research based largely on self-report or CD4 testing suggested that most migrants living with HIV were infected prior to migration, but recent investigations from Europe utilizing more robust methods indicate that high proportions of migrants acquire HIV infection after migration (Ross et al., 2018). For example, one study examining HIV acquisition among migrants at 57 clinics in nine European countries diagnosed in the preceding 5 years estimated that 63% of patients acquired HIV after migration (Alvarez-Del Arco et al., 2017). Of 2009 participants, 46% were MSM and a third originated from sub-Saharan Africa and Latin America and the Caribbean, respectively (Alvarez-Del Arco et al., 2017). Similarly, a study of migrants from sub-Saharan Africa estimated that 49% of participants in their study acquired HIV while living in France. Data from North America regarding place of acquisition are similar, though more limited: findings from a large, nationally representative transmission network analysis in the USA suggest that the majority of transmission partners of male foreign-born persons (63%) were born in the USA, whereas the majority of transmission partners of female foreign-born persons (57%) were born in their same world region (Desgrées-du-Loû et al., 2015). Other smaller US studies also provide evidence of local HIV transmission (Dennis et al., 2017; Kerani et al., 2017; Wiewel et al., 2015). For our study, while it is probable that those diagnosed during their arrival year were infected elsewhere, these data cannot determine the location where transmission occurred. Nevertheless, our results did identify that most of these diagnoses occurred within the first 5 years post-migration, which reinforces the argument that HIV testing not only should occur at immigration, but also should be regularly repeated within the first 5 years post-migration. This finding also emphasizes a potential need for HIV prevention services within this time period, including the use of HIV PrEP.

Second, our findings shed light on some clinical indicators and their relationship to HIV diagnosis in Ontario. To explain further, clinical evaluation helps determine whether a patient indicates the appropriate risk factors to be tested for HIV. Often these decisions are made based on patient-reported risk factors, which are subject to recall and social desirability bias (Kelly et al., 2013). A history of other infectious diseases that relate to HIV risk is an independent metric to determine whether HIV prevention, such as HIV PrEP, is indicated. Our findings align with international data, reinforcing that, in Ontario, a diagnosis of gonorrhea, chlamydia, and/or syphilis among MSM is an objective risk indicator for HIV acquisition, which warrants the use of HIV PrEP. Over 20% of the new diagnoses we reviewed among MSM had been previously diagnosed with one of these STIs. These findings thus support the current Canadian guidelines on PrEP, which strongly recommend PrEP for MSM who are diagnosed with bacterial STIs (Tan et al., 2017). While clinical charts were reviewed for PrEP referral and prescriptions, the data were not complete enough to offer any insights into PrEP offer, refusal, or failures.

What our findings add to the extant literature is the importance of considering a prior diagnosis of hepatitis C as an objective risk indicator for HIV acquisition among heterosexual men and women, as in the reviewed charts there were 12.7% and 10% rates of hepatitis C infection prior to HIV diagnosis, respectively. While this finding may not be surprising, given hepatitis C coinfection is not uncommon in people living with HIV and both infectious diseases are often transmitted by needle sharing in people who use injection drugs (Platt et al., 2016), neither the current Canadian nor US PrEP guidelines recommend PrEP for persons with previous hepatitis C diagnoses (Tan et al., 2017; Centers for Disease Control and Prevention, US Public Health Service, 2021). In light of shifting and varied HIV epidemic factors among different populations, identifying nascent data on risk factors that may precede HIV acquisition in groups other than MSM is important for overall HIV prevention efforts. Consideration should also be made to increase the threshold for offering PrEP to heterosexual men and women, particularly those with a recent hepatitis C diagnosis and/or who report engaging in injection drug use.

Third, our data shed some light on the robustness of HIV testing programs in Ontario by identifying whether those being diagnosed had missed earlier opportunities for testing and prevention. Even among those who were Canadian-born and those in Canada for more than 10 years, a quarter to a half had no history of HIV testing. While recent immigrants had a higher risk of AIDS diagnosis (33%), those who were Canadian-born still accounted for 20% of concurrent HIV/AIDS diagnoses. This is evidence that persons from all groups at risk for HIV do not, or cannot, engage in HIV testing or prevention activities. Further outreach is necessary to engage people at risk for HIV in testing and prevention. At the health systems level, one tangible strategy to help identify persons with undiagnosed HIV infection more rapidly would be the promotion of routine HIV testing for all populations, rather than relying on persons opting into HIV testing based on their self-perceptions of HIV risk. Such an approach might normalize HIV testing as a recommended routine health screening activity, similar to other conditions like diabetes, dyslipidemia, and cancer. Establishing clinical guidelines that encourage clinicians to offer testing in higher-risk populations may also help increase HIV testing among persons with delayed undiagnosed HIV infections. Knowing that many risk factors for HIV acquisition are socially stigmatized, a generalized testing approach might lower the risk of delayed diagnosis. Clinicians should also remember to routinely screen persons for STIs as well, and to use these diagnoses as an indicator condition for HIV testing and prevention services (like PrEP). Finally, clinicians should routinely actively offer PrEP to their patients with risk factors for HIV, as recommended in guidelines and as we have identified herein. In other words, clinicians should not wait for patients to request PrEP, but they should initiate conversations about PrEP and should then be willing to prescribe it to their patients.

### Limitations

Our findings must be interpreted considering certain limitations. First, our data collection was restricted to only three cities in Ontario—two of which (Ottawa, Hamilton) are medium-sized and one of which (Toronto) is the largest city in Canada. Differences related to immigration and prior hepatitis C diagnosis might change in smaller cities. These findings nevertheless identify useful information for medium-sized and large cities in Ontario. Second, our data collection was restricted to information that had already been collected at the time of HIV diagnosis. While this made the information contemporaneous, it also restricted the data to known risk factors from the time of diagnosis. Other yet-to-be determined risk factors may exist and would be outside this analysis. Third, there was a moderate amount of missing data in each category—again based on what was collected at the time of diagnosis. More complete records would potentially have changed some of our findings. Finally, the study does not include an HIV-negative comparison group and therefore cannot provide insight into independent predictors of HIV diagnosis (i.e., STBBI rates or HIV testing rates in those diagnosed with HIV as compared with their counterparts not diagnosed with HIV).

## Conclusion

The results of this study show (1) MSM with a recent diagnosis of HIV were likely to have a history of syphilis, gonorrhea, or chlamydia; (2) heterosexual men and women as compared with MSM had a greater likelihood of hepatitis C infection; and (3) immigrants diagnosed with HIV were more likely to be diagnosed within the first 5 years of migration.

High-income countries have the capacity to reach the UNAIDS 95-95-95 goals and effectively end local HIV transmission. However, accomplishing this will require specific efforts among those at risk for HIV who need access to testing, treatment, and prevention modalities. This study expanded beyond typical surveillance metrics to include STBBI diagnoses and immigration information. These indicators are key to targeting individuals for HIV testing and prevention services. As HIV transmission declines in high-income countries, it will be necessary to employ targeted strategies to ensure these decreases are experienced by all key populations. Without such efforts for all populations, it is unlikely that the UNAIDS 95-95-95 targets will be reached for 2030. Continuing efforts are necessary to disaggregate data and further understand the multiple simultaneous HIV epidemics. Moreover, these data should help us better understand and intervene for members of each affected subpopulation. By taking these actions, true and dramatic reductions in HIV transmission may be possible for all.

## Contributions to knowledge

What does this study add to existing knowledge?

- Ontario regularly reports on HIV surveillance metrics but does not include key indicators that may help better identify populations at risk for HIV and missed opportunities for HIV testing and prevention.
- This study expands on existing knowledge by further elucidating the role of migration in the local HIV epidemic, those presenting late to HIV testing, and co-infections with STBBIs.

What are the key implications for public health interventions, practice, or policy?

- This analysis shows that STBBI co-infections differ by key population and can likely help better identify those at risk for HIV, thereby helping to target HIV testing and prevention measures to those at risk.
- Further, early access to HIV testing post migration is critical to identify those who already know their status and those who are undiagnosed. An offer of repeat HIV testing and prevention services should be made for those whose HIV risk may persist or increase post migration.

## Data Availability

Data available upon request.

## Code availability

Available upon request.
